# Engineering Nano/Microscale Chiral Self-Assembly in 3D Printed Constructs

**DOI:** 10.1007/s40820-023-01286-0

**Published:** 2023-12-18

**Authors:** Mohsen Esmaeili, Ehsan Akbari, Kyle George, Gelareh Rezvan, Nader Taheri-Qazvini, Monirosadat Sadati

**Affiliations:** 1 Department of Chemical Engineering, University of South Carolina, Columbia, SC 29208 USA; 2 TA Instruments, Waters LLC, New Castle, DE 19720 USA; 3 Biomedical Engineering Program, University of South Carolina, Columbia, SC 29208 USA

**Keywords:** Directed chiral self-assembly, Cellulose nanocrystals, Bioinspired nanocomposite, 3D printing, Rheology

## Abstract

Helical hierarchy found in biomolecules like cellulose, chitin, and collagen underpins the remarkable mechanical strength and vibrant colors observed in living organisms. This study advances the integration of helical/chiral assembly and 3D printing technology, providing precise spatial control over chiral nano/microstructures of rod-shaped colloidal nanoparticles in intricate geometries. We designed reactive chiral inks based on cellulose nanocrystal (CNC) suspensions and acrylamide monomers, enabling the chiral assembly at nano/microscale, beyond the resolution seen in printed materials. We employed a range of complementary techniques including Orthogonal Superposition rheometry and in situ rheo-optic measurements under steady shear rate conditions. These techniques help us to understand the nature of the nonlinear flow behavior of the chiral inks, and directly probe the flow-induced microstructural dynamics and phase transitions at constant shear rates, as well as their post-flow relaxation. Furthermore, we analyzed the photo-curing process to identify key parameters affecting gelation kinetics and structural integrity of the printed object within the supporting bath. These insights into the interplay between the chiral inks self-assembly dynamics, 3D printing flow kinematics and photo-polymerization kinetics provide a roadmap to direct the out-of-equilibrium arrangement of CNC particles in the 3D printed filaments, ranging from uniform nematic to 3D concentric chiral structures with controlled pitch length, as well as random orientation of chiral domains. Our biomimetic approach can pave the way for the creation of materials with superior mechanical properties or programable photonic responses that arise from 3D nano/microstructure and can be translated into larger scale 3D printed designs.

**Supplementary Information:**

The online version contains supplementary material available at 10.1007/s40820-023-01286-0.

## Highlights

- To precisely engineer complex helical hierarchies at nano/microscales, reactive inks with chiral nematic anisotropy are designed for 3D printing.
- The phase transformations and chiral evolution in response to parallel and orthogonal shear forces are meticulously investigated to finely adjust the 3D printing parameters for programming oriented chiral assemblies.
- The interplay between chiral relaxation dynamics and photo-polymerization kinetics is finely tuned to enable well-controlled chiral reformation, while simultaneously ensuring high print quality.

**Supplementary Information:**

The online version contains supplementary material available at 10.1007/s40820-023-01286-0.

## Graphical abstract

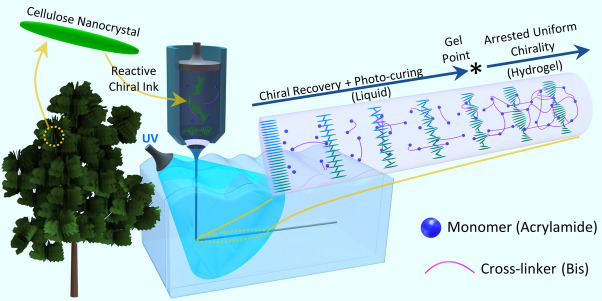

## Introduction

Advancing technology in applications such as health, energy, soft robotics, pharmaceuticals, and additive manufacturing is extensively involved in designing complex fluids at the scale of building constituents [1, 2]. Formulating complex fluids for additive manufacturing processes, however, requires a thorough understanding of structure-to-rheology and rheology-to-flow relations [3]. The flow properties of complex fluids become particularly important in the fabrication of functional structures in multiple length scales [4]. One of the strategies for fabricating hierarchical structures is exploiting the spontaneous self-assembly of materials, which requires components to be mobile in the fluid phase for self-constructing ordered structures [5].

Additive manufacturing techniques expanded material fabrication toward more flexible and versatile methods. However, most soft materials used in 3D printing are adapted from traditional techniques that constrain achieving hierarchies at smaller scales than the voxel level. For controlling the microstructure of 3D printed materials, synthesized complex fluids can be designed to synergistically couple the assembly pathway with the complex flow condition in 3D printing [6–8]. Direct ink writing (DIW), as an extrusion-based 3D printing technique, has been widely used to locally direct the material properties during 3D printing [9–12]. The flow inside the DIW's nozzle guides the self-assembly of anisotropic materials that has been widely used to enhance mechanical properties or induce anisotropic stimuli response in 3D printed constructs [13–17]. To analyze flow-induced self-assemblies, rheological measurements are commonly combined with other techniques, such as numerical simulations [18], in situ characterization [19], microfluidics [20], optical rheology [21], small angle light scattering [22, 23], and light spectroscopy [24].

Among anisotropic complex fluids are liquid crystals that can thermodynamically appear in different structures at stationary conditions, such as nematic, smectic, and chiral/cholesteric phases [25]. The orientation of liquid crystalline phases can be directed during DIW to program the anisotropic properties in printed materials [21, 26, 27]. Patterned liquid crystalline phases that respond to external stimuli such as temperature [28], light [29], humidity [30], magnetic fields [31], and electric field [32] tremendously expand toward soft robotics and actuation applications. The arrested structure in 3D-printed liquid crystalline materials, however, is limited to the flow-induced nematic phases that were obtained by in situ polymerization during DIW [33]. Here, flow analysis is essential to control the alignment order of nematic mesophases, and therefore, program material properties.

By employing chiral nematic inks during DIW process, in our previous work, we have shown the feasibility of achieving sub-voxel control over the 3D chiral (helical) self-assembly, confined within the cylindrical geometry of extruded filaments [34]. The inks were designed based on aqueous suspensions of cellulose nanocrystals (CNC), which are known to form chiral nematic nano/microstructures [35–39]. Chiral nematic describes an organized structure in which its elements are twisted relative to neighboring components, resulting in a periodic chirality characterized by a distinct pitch length. Nevertheless, the chiral assembly of CNC takes place at low concentrations in aqueous solutions, resulting in a liquid ink with low viscosity that displays a biphasic structure composed of isotropic and randomly distributed chiral nematic domains [19]. To address this challenge, photo-curable additives were incorporated into the chiral inks to enhance the viscosity post-polymerization and arrest the self-assembled nano/microstructure within a cross-linked polymer network. Here, in this study, we delve into the materials properties and processing parameters, introducing an engineering approach to modulate the chiral assembly while maintaining the print quality and consistency. We have utilized various complementary flow characterization techniques to understand the shear-induced structural dynamics and post-flow relaxation of the reactive chiral inks. We specifically conducted orthogonal superposition rheometry and in situ optical shear rheometry at constant shear rate conditions to accurately determine the impact of shear flow forces on the chiral nano/microstructures. Additionally, the optical shear rheometry and our laboratory-built microfluidic set-up were used to directly monitor flow-induced alignment of CNC particles and their post-flow relaxation with polarized optical microscopy (POM). They were performed, respectively, under constant shear rates and conditions that simulate the DIW process. Following ink deposition, the photo-polymerization and subsequent gelation of chiral inks locks in the arrangement of the CNC particles. Hence, it is crucial to adjust the gelation kinetics to provide sufficient time for the chiral structures to recover before the gelation takes place. To quantify the gelation time, the UV curing rheometry was performed on various inks with different CNC:Additive ratios and at different UV intensities. However, to support the printed filament until the photo-polymerization is complete, inks are printed within supporting baths of yielding microgels. Therefore, the photo-polymerization kinetics was further optimized for reactive inks deposited in the supporting bath by exploring the effect of monomer, crosslinker, and UV intensity. Understanding the interplay between inks structural dynamics, 3D printing flow kinematics, and polymerization kinetics provided key parameters to guide the arrangement of CNC particles in the 3D printed constructs, from uniform nematic to 3D concentric chiral nano/microstructures with tunable pitch length as well as randomly oriented chiral domains.

This study presents a practical biomimetic approach that harnesses 3D printing to create materials with distinct properties inherent to their tailored nano/microstructures, such as programmable photonics responses (dynamic colors) and enhanced mechanical properties (fracture resistant), which can be transferred to larger-scale intricate printed designs. This expands the horizons of 3D printing material technologies, propelling us into a new era of materials with unique properties for photonic metamaterials, smart textiles, and biomedical applications.

## Experiments

### Materials

CNC was supplied by CelluForce (Montreal, Quebec, Canada). Acrylamide (AAm) as a monomer, and N,N’-methylene-bisacrylamide (Bis) as a cross-linker were used as received from Alfa Aesar. 2-Hydroxy-2-methyl propiophenone (Irgacure 1173) as a photo-initiator was purchased from TCI. Dry powder of commercial Carbopol 980 NF was kindly provided by Lubrizol (Cleveland, OH, USA). Milli-Q water was used to prepare CNC suspension and Carbopol gel. Quartz capillary tubes were supplied from VITROCOM (Mountain Lakes, NJ, USA) with inner and outer diameters of 400 and 550 µm, respectively.

### Sample Preparation

#### Chiral Inks

Photo-curable inks were prepared by dispersing AAm and Bis in aqueous CNC suspensions, followed by probe sonication. The initial non-sonicated CNC suspensions were prepared as previously described [19]. Briefly, dry CNC particles at 7 wt% were added to Milli-Q water and vigorously dispersed using a homogenizer (Fisherbrand 850 Homogenizer, Fischer Scientific, Newington, NH, USA) in five two-minute cycles. The same process was repeated after resting CNC suspensions overnight to ensure full dispersion of CNC particles in water. Subsequently, AAm and Bis were added to the prepared 7 wt% of CNC suspension in batches of 10 gr, as described in Table 1. In all samples, Irgacure 1173, photo-initiator, was added at the constant concentration of 0.5 wt% with respect to the total amount of additives (AAm and Bis). Batches of 10 gr were vortexed in 20 mL vials until homogeneity was confirmed by the naked eye. Afterward, samples were sonicated at the constant input energy density of 5,000 J/gr-of-CNC using a 120-W Fisherbrand sonicator (Fischer Scientific, Newington, NH, USA) equipped with a 3 mm probe at the amplitude of 80%. Following the procedure, five chiral inks with different compositions of monomer and crosslinking ratios were prepared. At the Bis:AAm ratio of 1:30, three inks made with CNC:Additive ratios (CNC:Add.) 1:2, 1:4, and 1:6, in which the code “Add” represents Additives composed of AAm, Bis, and photo-initiator. To investigate the effect of crosslinking ratio, at the constant (CNC:Add.) ratio of 1:4, the Bis:AAm ratio in the additive was varied (Bis:AAm = 1:15, 1:30, and 1:60). In Table 1, therefore, samples were introduced as Add(X)-Bis(Y), where X identifies the ratio of additives to CNC and Y represents the ratio of Bis to AAm in the inks.

**Table 1:** Composition of different samples at a total weight of 10 gr

Sample’s abbreviation	CNC:Additive ratio	Bis:AAm ratio	7 wt% of CNC suspension (gr)	AAm (gr)	Bis (mg)	CNC content (wt%)
Add(2)-Bis(1/30)	1:2	1:30	8.771	1.188	39	6.14
Add(4)-Bis(1/15)	1:4	1:15	7.812	2.050	136	5.47
Add(4)-Bis(1/30)		1:30		2.116	70	
Add(4)-Bis(1/60)		1:60		2.151	35	
Add(6)-Bis(1/30)	1:6	1:30	7.042	2.862	95	4.92

#### Supporting Bath

Carbopol microgels for supporting baths were prepared at the concentration of 0.5 wt% by dispersing Carbopol 980 NF powder in Milli-Q water using an overhead stirrer (Fisher Scientific, Newington, NH, USA). When no more visible clusters were observed, NaOH 1 M at 600 µL/gr-of-Carbopol was slowly added to form jammed microgels of Carbopol, followed by further homogenization using the previously described homogenizer. Trapped bubbles were removed using a planetary centrifugal DAC 330-100 SE speed mixer (FlackTek SpeedMixer, Landrum, SC, USA) at the speed of 3,000 rpm for three minutes.

### Polarized Optical Microscopy (POM)

A ZEIS Axioscope 5 (Oberkochen, Germany) was used for all POM analyses in the transmission mode. Stationary measurements were carried out using laboratory-made sample cells, made of two cover glasses and 200 µm of spacers in between. POM images were collected after one hour of loading inks into sample cells to ensure the stability of formed structures.

### Rheological Measurements

All rheological experiments were performed using a Discovery HR-2 (TA instruments, Delaware, USA). For flow sweep tests on CNC-based inks, a 40 mm aluminum cone geometry and a solvent trap were used at 25 °C. To obtain viscosity plots, CNC-based inks were sheared under steady-state shear rates from 0.01 to 1000 s^−1^. Before each flow measurement, inks were pre-sheared at the first data point (i.e., the shear rate of 0.01 s^−1^) for 120 s to ensure a steady-state rotational flow is achieved since the beginning of the test. Following our previous methodology [19], the sampling time for each data point was set at 30 s to ensure reaching an equilibrium state under the applied shear rate. Orthogonal superposition (OSP) measurements were carried out by using Discovery HR-30 (TA instruments, Delaware, USA) and a special double wall Couette geometry to simultaneously impose rotational and axial deformation. The oscillatory deformation was applied perpendicular to the rotational steady-state shear flow. The angular frequency of the orthogonal oscillation varied from 100 to 0.1 rad s^−1^ at the normal strain of 2%. The shear rate of the rotational steady-state shear flow varied from 0.01 to 1,000 s^−1^. For transient stress relaxation measurements, inks were initially pre-sheared at the shear rate of 37 s^−1^ for one minute, followed by an immediate decrease in shear rate to 0.01 s^−1^. Recorded shear stresses were normalized with respect to their minimum value. To determine gelation times, the UV curing accessory on the Discovery rheometer equipped with an OmniCure S2000 spot UV curing system (Excelitas Technologies, Waltham, MA, USA) was used. Using 20 mm stainless steel geometry and quartz Peltier, oscillation fast sampling tests were performed using a 20 mm plate geometry at the angular frequency of 1 rad s^−1^ and the shear strain of 0.1% for 420 s, during which the UV light was irradiated on ongoing oscillating samples at the 300th second. The flow sweep test on Carbopol microgel was performed using a 40 mm stainless steel plate geometry at 25 °C with a gap size of 750 µm. The shear rate was varied from 1000 to 0.001 s^−1^, with a sampling time of 60 s to ensure steady-state conditions at every measured data point.

### Optical Rheology

Optical rheology analysis was performed by coupling the ZEISS microscope with a Linkam CSS450 Optical Rheology Stage (Surrey, UK). The stage was mounted onto the microscope with the circular flow direction at the observation radius of 7.5 mm oriented at 45° with respect to cross-polarizer axes. The gap size of the Linkam stage was set at 400 µm. To obtain POM images of steady-state shear flows at different shear rates, a sufficient time was allowed to elapse until a steady-state structure was formed. This time was estimated using real-time POM observation and the value of the inverse shear rate. Time-series POM images were captured by stopping steady-state flows at 37 s^−1^ and instantaneously recording videos of structural transitions.

### Microfluidic Analysis

A microfluidic set-up was designed in the laboratory to assess the structural changes that occur following the cessation of flow, as schematically shown in Fig. 3a. In this measurement, samples were made to flow through quartz capillaries with an inner diameter of 400 µm, which were aligned at 45° with respect to the cross-polarizer axis. The flow rates in the capillary were chosen to be equivalent to the actual flow rate during the 3D printing and were regulated using a syringe pump (Harvard Apparatus, Holliston, MA). To minimize the creeping flow, a valve was used to shut down the flow at the entrance of the capillary tubes. POM images were captured using two types of light sources: a white light source and a monochromic light, filtered by a 500 nm bandpass light filter (FB500-10, Thorlabs, λ = 500 nm). The detailed analytical and experimental analysis of birefringence value and retardation length measurements using the monochromic light are provided in the supporting information.

The optical contrast parameter, defined as $\frac{{I_{\max } - I_{\min } }}{{I_{\max } + I_{\min } }}$, was measured following the method described by Haywood et al. [24]. Samples flowed through quartz capillaries under monochromic light (500 nm) in a cross-polarized configuration. Two separate experiments were conducted for each sample, with the flow direction oriented at 45° and 0° relative to the analyzer axes, in order to record the *I*_max_ and *I*_min_ values, respectively. These values were obtained by measuring the average RGB intensities immediately after flow cessation for one hour. All experiments were conducted with equal exposure time for consistency.

### Direct Ink Writing (DIW)

Photo-curable inks were loaded into 5 mL HSW syringes and mounted onto a DIW 3D printer (System 30 M, HYREL 3D, Norcross, GA, USA). Blunt needles with an inner diameter of 410 µm (Gage 22) and a length of 1½ inches were used as DIW's nozzles. Samples were printed in a rectilinear pattern within supporting baths of Carbopol microgels, while simultaneously being exposed to different intensities of UV light, directed from the OmniCure S2000 spot UV curing system (Excelitas Technologies, Waltham, MA, USA), using a UV light guide. The UV absorption of the supporting bath was measured using the detector plate of the Silver Line UV-meter (UV-DESIGN, Germany) immersed into the supporting bath at the depth equivalent to the printing depth. Infill density of 30%, translation speed of 1 mm s^−1^, and flow multiplier of 3 were used as identical printing conditions throughout this study. Further UV curing was assured using a UV bench lamp (365 nm, Analytik Jena, Jena, Germany) for one hour.

In our DIW process, the average shear rate, experienced by the inks inside the DIW nozzle, was calculated to be approximately 37 s^−1^ using the formula $\dot{\gamma }_{{{\text{ave}}{.}}} = \frac{3n + 1}{{2n + 1}}\frac{{2V_{{{\text{ave}}{.}}} }}{r}$, where *n*, *V*_ave._, and *r* are power law index, average flow velocity (3 mm s^−1^), and the nozzle radius (205 µm), respectively [40, 41]. The power law index of 0.63 was used to calculate the average shear rate. This value was obtained by averaging the indices from fitting the power law model to the second shear thinning part of the flow curve (regime III) for three different ink compositions (Fig. S1). Our flow-induced birefringence analysis has shown that at the average flow velocity of 3 mm s^−1^, CNC particles align themselves along the flow direction, adopting pseudo nematic configuration [19].

### Scanning Electron Microscopy

Single filaments were printed using ink Add(4)-Bis(1/30) under optimal printing conditions within a supporting bath. After printing, the filaments were allowed to dry. Subsequently, the dried filaments were carefully snapped, and to prevent charging effects, they were sputter-coated with a layer of gold/palladium for 120 s. The morphology of the fracture surface of the snapped filaments was then examined using a field emission scanning electron microscope (FE-SEM, Zeiss Gemini500).

## Results and Discussion

### Effect of Shear Flow on the Chiral Nematic Structure

**Fig. 1:**
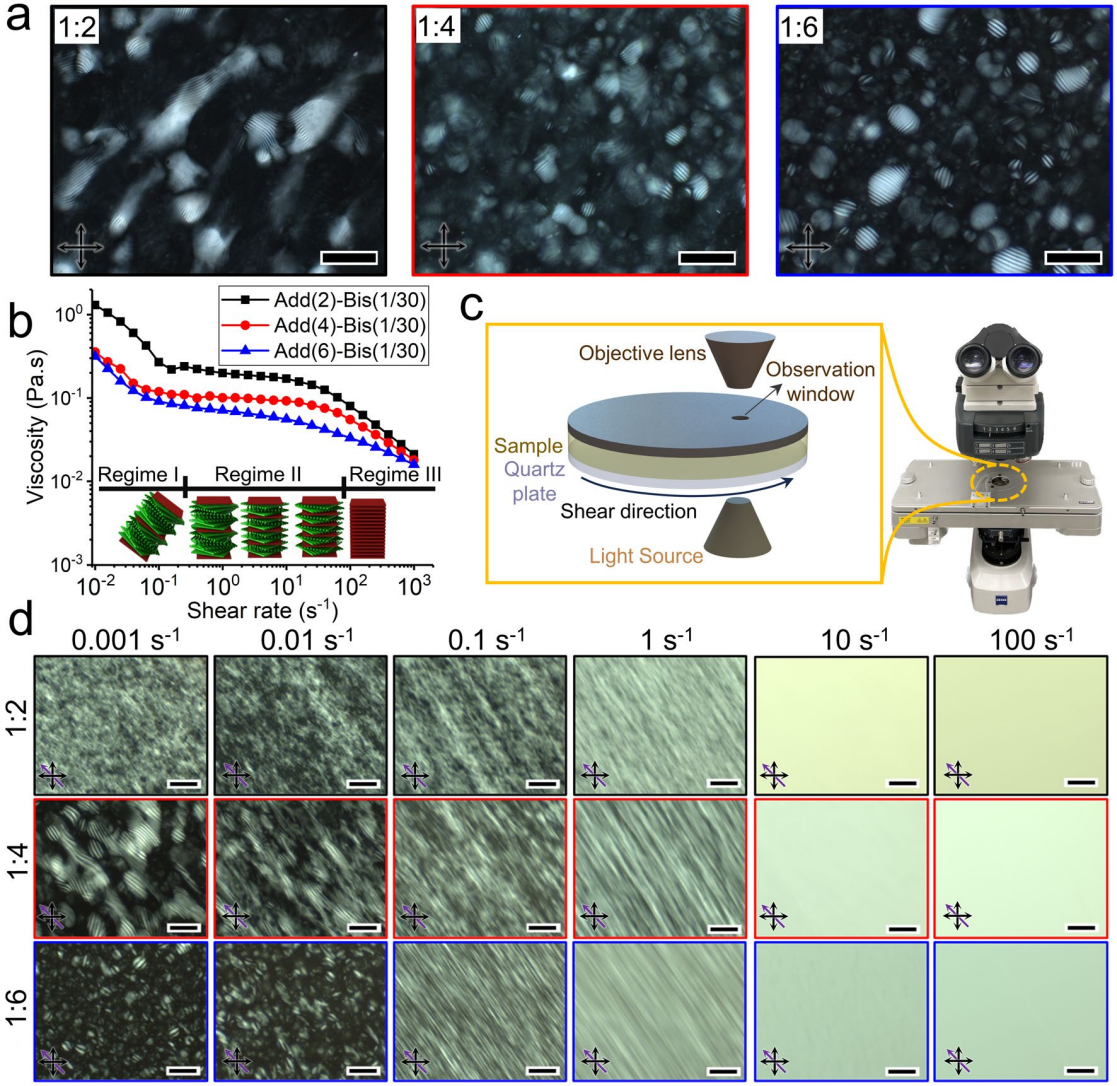
Structural evolution of chiral nematic inks under the simple shear flow. **a** POM images of photo-curable inks with different CNC:Add. ratios and a constant cross-linker ratio of 1:30 at the stationary condition. **b** Viscosity as a function of shear rate for different CNC:Add. ratios at a constant cross-linker ratio of 1:30. The schematic demonstration of three characteristic flow regimes of chiral nematic structure from domain rotation in regime I (low shear rates), uncoiling in regime II (intermediate shear rates), and eventually the individual alignment of CNC particles in regime III (high shear rates). **c** A schematic and a photograph presenting the Linkam optical rheometry stage installed on a polarized optical microscope. The schematic inset highlights the rotation of the bottom quartz plate, which precisely applies a preset shear rate at a specified distance from the center (7.5 mm), while the Zeiss microscope captures POM images through the observation window. **d** POM images of optical rheology measurements on inks with different CNC:Add. ratios using the Linkam stage at 400 µm gap size and various shear rates. Scale bars are 100 µm

To achieve the chiral nematic assembly of the CNC particles during 3D printing, it is crucial to carefully analyze the interaction of flow forces with the microstructure of chiral inks. The chiral assembly of CNC particles is formed at low concentrations, resulting in chiral inks that exhibit liquid-like behavior and remarkably low viscosity (~ 1 Pa s [19]). This characteristic makes it difficult to achieve a self-supporting feature of the printed filament during the DIW process. To address this issue, we propose the incorporation of photo-curable additives of AAm and Bis into a chiral nematic aqueous suspension with the maximum CNC concentration within the critical biphasic range (7 wt%) [34]. This will result in a cross-linked network upon UV light exposure, improving the shape-fidelity of printed filaments after DIW. POM images taken at the stationary condition of inks with different CNC:Add. ratios show the fingerprint patterns, indicative of the chiral nematic assembly of CNC particles (Fig. 1a). Additionally, all inks contain isotropic (dark regions in Fig. 1a), and anisotropic (chiral nematic domains with fingerprint patterns in Fig. 1a) phases, indicating biphasic behaviors at the stationary condition. Due to the inherently weak repulsive electrostatic interaction among CNC particles [42], the chiral arrangement of CNC particles can be readily disturbed by the flow forces inside the DIW's nozzle [19, 21]. In our previous work [34], we analyzed the viscosity trend of our chiral nematic inks and proposed a hypothesis on helical uncoiling. In this study, we provide detailed structural analysis under various shear rates using rheo-optics analysis and orthogonal superposition rheometry. Under the steady-state rotational shear flow, viscosity trends of all reactive chiral inks exhibit a characteristic three-regime flow behavior of lyotropic liquid crystals [19], with similar threshold shear rates (Fig. 1b).

The viscosity plots of the chiral inks can be divided into three distinct regimes [23, 43]. The first regime (regime I) is identified by a shear-thinning behavior at low shear rates, which is attributed to the orientation of chiral domains along the rotational flow direction (Fig. 1b). By using the Linkam CSS450 Optical Rheology Stage (rheo-optics, Fig. 1c), this domain orientation is confirmed at steady-state shear rates of 0.001 and 0.01 s^−1^ (Fig. 1d). As the shear rate increases, viscosity plots exhibit a Newtonian behavior in regime II, which correlates with the uncoiling of the already aligned chiral nematic domains (Fig. 1b). Optical rheology images at steady-state shear rates of 0.1, 1, and 10 s^−1^ demonstrate the gradual uncoiling process, as the periodic pitch length of fingerprint patterns gradually decreases to the point where aligned fingerprint patterns fade into uniform birefringence bright patterns (Fig. 1d). At sufficiently high shear rates, the second shear-thinning behavior, regime III, occurs, where individual CNC particles are fully aligned along the flow direction and form a pseudo-nematic state (Fig. 1b). Due to the gradual aspect of chiral uncoiling, the transition from regime II to III smoothly occurs at shear rates above 10 s^−1^. This flow-induced pseudo-nematic anisotropy results in the emergence of birefringence bright colors at steady-state shear rates above 10 s^−1^ (Fig. 1d). Interestingly, even though chiral inks with higher monomer contents exhibit a larger fraction of isotropic domains under stationary conditions (Fig. 1a) and low shear rates (shear rates of 0.001 and 0.01 s^−1^ in Fig. 1d), increased shear forces result in the remarkable transformation of their structures into fully anisotropic monodomains with uniformly aligned chiral nematic structures. This suggests the potential of achieving fully ordered chiral structures in 3D printed filaments, independent of the biphasic behavior of chiral inks at stationary conditions. Based on this structural analysis under primary shear flows and considering the average shear rate in our DIW approach (37 s^−1^ which falls at the beginning of regime III), we can assume that the CNC particles predominantly adopt a pseudo-nematic structure inside the DIW's nozzle.

**Fig. 2:**
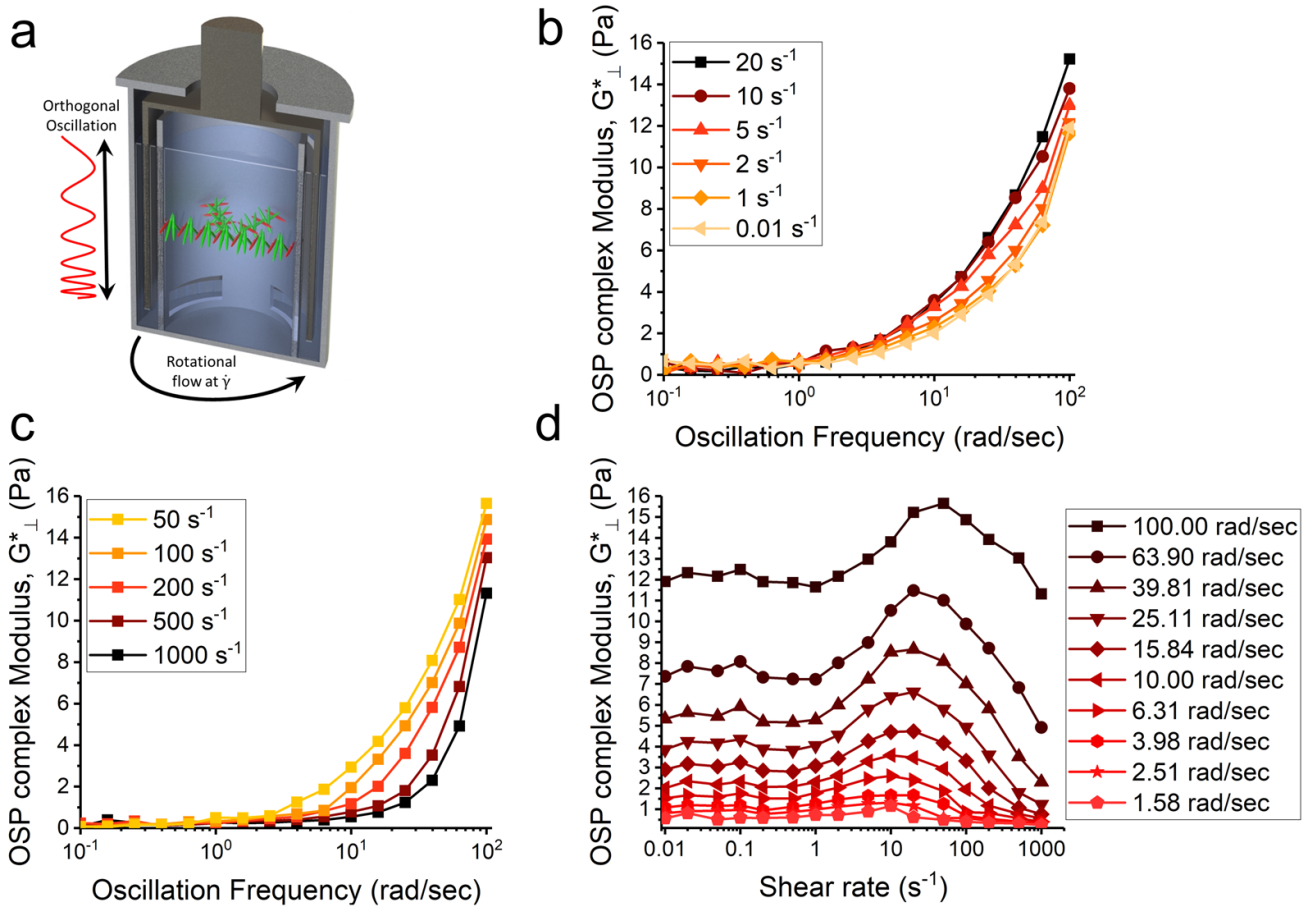
OSP measurements on sample Add(4)-Bis(1/30) at shear rates from 0.01 to 1,000 s^−1^ and oscillation frequency from 0.1 to 100 rad s^−1^. **a** schematic illustration of OSP measurements on chiral assembly of CNC particles. **b** Frequency dependence of G*_⊥_ at shear rates from 0.01 to 20 s^−1^ (within Regime I and II), and **c** from 50 to 1000 s^−1^ (within Regime III). **d** Effect of applying rotational shear rate on G*_⊥_ at different oscillation frequencies

In addition to the observation of the three distinct regimes in the rotational direction (Fig. 1b), the uncoiling process can also be analyzed in the perpendicular direction, where the normal forces are applied. Previous studies have shown that in high-viscosity chiral nematic liquid crystals, where the axial force falls within the detection range of conventional rheometers, the normal stress difference (N_1_) under simple shear flows shows two transition thresholds that closely match the transitions between three regimes in viscosity trends [43–49]. Our recent study on highly concentrated hydroxypropyl cellulose (HPC) solutions (63 wt%) with chiral nematic structures has revealed that the transition from regime II to regime III is linked to the emergence of a maximum in N_1_ plots [50]. However, measuring N_1_ in low-viscosity liquid crystals, such as CNC suspensions, is challenging due to instrumental limitations. While rheological properties of complex fluids are typically assessed using either rotational shearing or oscillatory deformation, the superposition rheological measurements, where rotational and oscillatory deformations are applied simultaneously, allows for a more comprehensive analysis of the flow-induced structural evolution. However, when the imposed oscillatory deformation is at the same direction as the rotational flow, called parallel superposition, strong coupling of measured moduli with the steady state shear flow complicates flow-induced structural analysis [51, 52]. Recent instrumental developments on orthogonal superposition rheology (OSP) allow performing oscillatory deformations perpendicular to primary steady-state shear flows, which separates measured rotational and oscillatory parameters. In this study, we leveraged this new technique to superimpose small-amplitude oscillation, orthogonal to the ongoing rotational shear flows, at various shear rates (Fig. 2a). These measurements to our knowledge represent for the first time the structural analysis of chiral structures uncoiling under the shear flow in the perpendicular direction.

OSP measurements are carried out on sample Add(4)-Bis(1/30) as a representative of our chiral nematic inks. As the rotational shear rate was varied, the OSP complex modulus, G*_⊥_ ($= \frac{{\sigma_{ \bot } }}{{\gamma_{ \bot } }}$, where σ_⊥_, and γ_⊥_ are normal stress and strain amplitude, respectively), exhibited a plateau behavior at low frequencies, while increasing at high frequencies (Fig. 2b, c). Within the regime I (shear rates < 1 s^−1^), G*_⊥_ is not affected by the primary shear flow, indicating no change in the interaction between CNC particles (Fig. 2b). This is consistent with the findings of Regime I (Fig. 1), where shear flow only orients chiral nematic domains without disturbing the helical assembly of CNC particles (Fig. 1d). Furthermore, it can be inferred that the rotation of chiral nematic domains does not induce the accumulation of excess energy within regime I.

As the shear rate increased up to 20 s^−1^, G*_⊥_ plots shifted upward at higher frequencies, suggesting an increase in stored energy caused by the disrupted helical assembly of CNC particles under the shear flow within regime II (Fig. 2b). We hypothesize that the out-of-equilibrium in-plane uncoiling of helical assembly under the steady-state rotational flow generates a mechanical response perpendicular to the helical axis, leading to higher G*_⊥_ values. Moreover, low values of G*_⊥_ within regimes I and II indicate that slow relaxation mechanisms, such as phase separation, are eliminated under the steady state shear flows. A reverse trend was observed with a further increase in the rotational shear rate within regime III. Specifically, there was a downward shift in the plots of G*⊥ at high frequencies, while the values remained constant at low frequencies (< 1 rad s^−1^) (Fig. 2c). This trend within regime III is consistent with previous reports on the decrease of N_1_ values due to the wagging motion of liquid crystalline directors [53, 54], indicating that CNC particles oscillate back and forth about the flow direction. Compared to lower shear rates, the plateau range of G*_⊥_ at low frequencies is expanded toward higher frequencies within regime III, indicating that strong shear flows eliminate faster relaxation mechanisms such as retwisting by restricted wagging motion of CNC particles (Fig. 2c).

The observed three distinct regimes of G*_⊥_ under rotational shear flows (Fig. 2d) show a strong correlation with the characteristic three-regime behavior observed in the rotational direction (Fig. 1). Additionally, when plotting G*_⊥_ against the shear rate at various frequencies, overshoots are observed in G*_⊥_ plots at oscillatory frequencies higher than 1.58 rad s^−1^, which is further amplified by increasing the oscillatory frequency (Fig. 2d). The shear rates at which the overshoot in G*⊥ plots were observed closely correspond to the average shear rate employed in our DIW approach (37 s^−1^). This chiral unwinding-induced stored energy is expected to be relieved after the ink deposition, where the shear flow stops. These observations are consistent with our previous findings on chiral nematic HPC solutions, where N_1_ plots displayed a plateau trend at low shear rates, followed by an initial increase toward a maximum and eventually a decreasing trend [50]. However, our current measurements capture the time scale during which the chiral director adapts itself to the new shear-induced condition to oscillate back and forth about the flow direction. Based on the relaxation time of twisting CNC particles, determined from the oscillatory frequency at which the overshoot was initiated, i.e., 1.58 rad s^−1^, it can be inferred that CNC particles require less than 4 s $\left( { = \frac{2\pi }{{1.58}}} \right)$ to adapt the shear-induced condition in the nozzle of the 3D printer. This implies that in our employed DIW approach (with a print speed of 1 mm s^−1^ and a flow multiplier of 3), chiral inks transition into the pseudo-nematic state after traveling 12 mm inside the nozzle, which is shorter than the length of the nozzle itself (1½ inches, equivalent to 38 mm). Consequently, a fully developed flow of the pseudo-nematic structure is established within the nozzle prior to the extrusion of the ink.

### Post-flow Dynamics of Chiral Nematic Structure

**Fig. 3:**
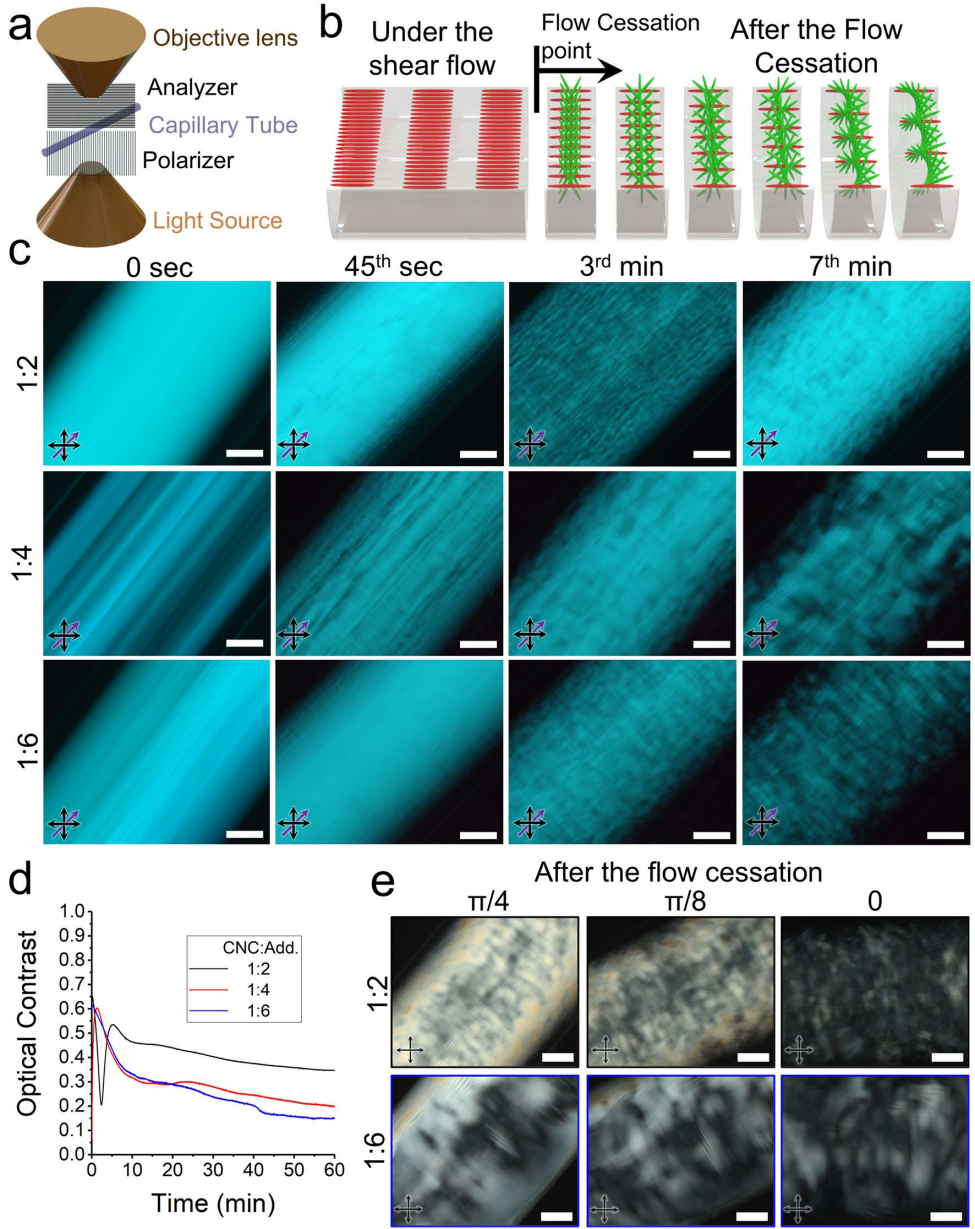
The structural evolution after the flow cessation. **a** Schematic presentation of the employed microfluidic setup, coupled with cross-polarized microscopy to in situ characterize the structural relaxation of capillary flows, oriented at 45°. **b** Schematic illustration of chiral nematic relaxation, confined within the cylindrical geometry of capillary tubes, after flow cessation. **c** Various time-series snapshots of different inks after flow cessation under POM using a monochromic light source. Black and purple arrows indicate the polarizer/analyzer and the flow direction, respectively. **d** The trend of optical contrast parameter after flow cessation for different CNC-based inks. **e** POM images of capillary tubes filled with different CNC-based inks one hour after flow cessation at different angles of flow direction with respect to the polarizer direction. Scale bars are 100 µm

According to our flow characterization, the average shear rate of 37 s^−1^ employed in the DIW process is expected to align the CNC particles along with the flow direction in the nozzle, leading to the formation of a monodomain pseudo-nematic structure. However, we have recently shown that this flow-induced structure is metastable and tends to release the excess stored energy and relax back to the ground state of chiral nematic structures after flow cessation [34]. Here, we aim to precisely determine the structural relaxation dynamics of reactive chiral CNC inks after printing and understand the effect of photo-polymerization kinetics on the arrangement of the CNC particles. To accomplish this goal, we performed microfluidic measurements to monitor the structural evolution immediately after stopping the Poiseuille flow through a quartz capillary with the same size as the DIW's nozzle (Fig. 3a). The microfluidic device was mounted on a polarized optical microscope equipped with two monochromic (Fig. 3c) and white light sources (Fig. S2). POM images captured using a monochromatic light source provide higher resolution of fingerprint patterns at earlier time frames, enabling the detection of chiral nematic structure formation. As discussed in the previous section, before the flow cessation, the flow-induced pseudo-nematic structures in the CNC-based chiral inks manifest as fully bright patterns (*t* = 0 s in Figs. 3c and S2) and birefringent patterns (Fig. S2) under monochromic and white light source, respectively. After the flow cessation, however, unidirectional fingerprint patterns emerged, indicating the formation of the uniformly aligned chiral nematic structure from a prior pseudo-nematic structure (Figs. 3c and S2). Moreover, the periodic characteristic pitch length of the appearing fingerprint patterns increased gradually after the flow cessation, indicating the smooth relaxation of chiral nematic assembly (Figs. 3b, c and S2). The rate at which the chiral structure recovers (relaxes) strongly depends on the ink composition; more additives led to the faster disappearance of birefringence patterns (Fig. S2) and earlier formation of fingerprint patterns (Figs. 3c and S2). Although the inks exhibited biphasic behavior, with a larger isotropic phase observed in those containing higher monomer content (Fig. 1a), fully chiral nematic structures formed shortly after the flow cessation stopped. This implies that our approach is capable of 3D printing uniformly aligned chiral nematic structures regardless of the biphasic structure at stationary conditions. Moreover, we can adjust the rate of chiral relaxation solely by modifying the ink composition, without the risk of creating isotropic domains. The phase separation, however, occurs through the nucleation-and-growth mechanism at later time frames after flow cessations, indicating the inks reach their equilibrium biphasic state at stationary conditions. As the CNC:Add. ratio decreases, isotropic phases appear earlier and grow to a greater extent (Figs. 3c and S2). This highlights that, based on the ink composition, it is not only necessary to allow for a minimum amount of time to pass for the recovery of chiral structures but also to take careful precautions to prevent phase separation in later timeframes.

The phase separation at later time frames after the flow cessation determines a critical upper limit for the time window within which the fully chiral nematic structure in the uniform concentric configuration forms. To understand the structural evolution of chiral inks after flow cessation, the orientation of liquid crystalline directors has been extensively studied, with the order parameter serving as a critical determinant in both theoretical and experimental investigations [22, 24, 55–61]. In our recent study, we employed image analysis to create alignment vector fields from snapshots after flow cessation and measured the order parameter accordingly [34]. Here, we show that optical contrast parameter determined using monochromic light can be used as an alternative quantitative optical approach to study the recovery of chiral structures after flow cessation (Fig. 3d).

The overall pattern observed in the optical contrast parameter following the cessation of flow signifies a gradual loss of orientation of CNC particles over time (Fig. 3d). The optical contrast of sample Add(2)-Bis(1/30), displays a minimum value shortly after the flow cessation (Fig. 3d). This sample initially exhibits second-order retardation colors (within the 550–1100 nm range, as illustrated in Fig. S3) under the shear flow. After the shear removal, birefringence colors with lower retardation lengths appeared gradually (Fig. S2), signifying the transition from the second order to the first order. In monochromic POM images (Fig. 3c), however, this transition decreases the light intensity (*I*_max_) around the third minute after the flow cessation, which is correlated to the periodic nature of the tan^−1^ function in Eqs. S1 and S2. As a result, the calculated values of optical contrast for ink Add(2)-Bis(1/30) reach a minimum during this transition. After this transition, however, the subsequent smooth decrease of *I*_max_ leads to the gradual decline of the optical contrast parameter. However, the optical contrast in this sample remains relatively stable one hour after flow cessation, indicating the recovered chiral nematic structure does not undergo significant structural transitions. In addition, analysis of POM images captured one hour after the flow cessation of sample Add(2)-Bis(1/30) indicates that most of the recovered chiral structure remains uniformly aligned with the flow direction. This is evident by the decrease in transmittance light intensity as the angle between the flow direction and polarizer axes diminishes (Fig. 3e). Also, the majority of sample Add(2)-Bis(1/30) confined inside the capillary tube remains anisotropic after one hour of the flow cessation, indicating the higher degree of alignment in this ink compared to other samples (Fig. 3e). However, in samples Add(4)-Bis(1/30) and Add(6)-Bis(1/30), the optical contrast plots exhibit a sharp decrease (Fig. 3d), indicating a quick orientational relaxation of CNC particles after the flow cessation (Figs. S4 and 3d). One hour after the flow cessation, the random alignment of chiral domains and the formation of isotropic regions are evident in the POM images captured from different flow directions relative to the analyzer axis (Fig. 3e). The tendency toward phase separation and domain disorientation is attributed to the decreasing trend of the anisotropic phase ratio as more additives are incorporated.

These observations regarding long-term structural changes after the flow cessation suggest that the chiral relaxation rate increases with the addition of more additives to the inks. However, this can also accelerate the undesired phenomenon of phase separation and biphasic formation.

**Fig. 4:**
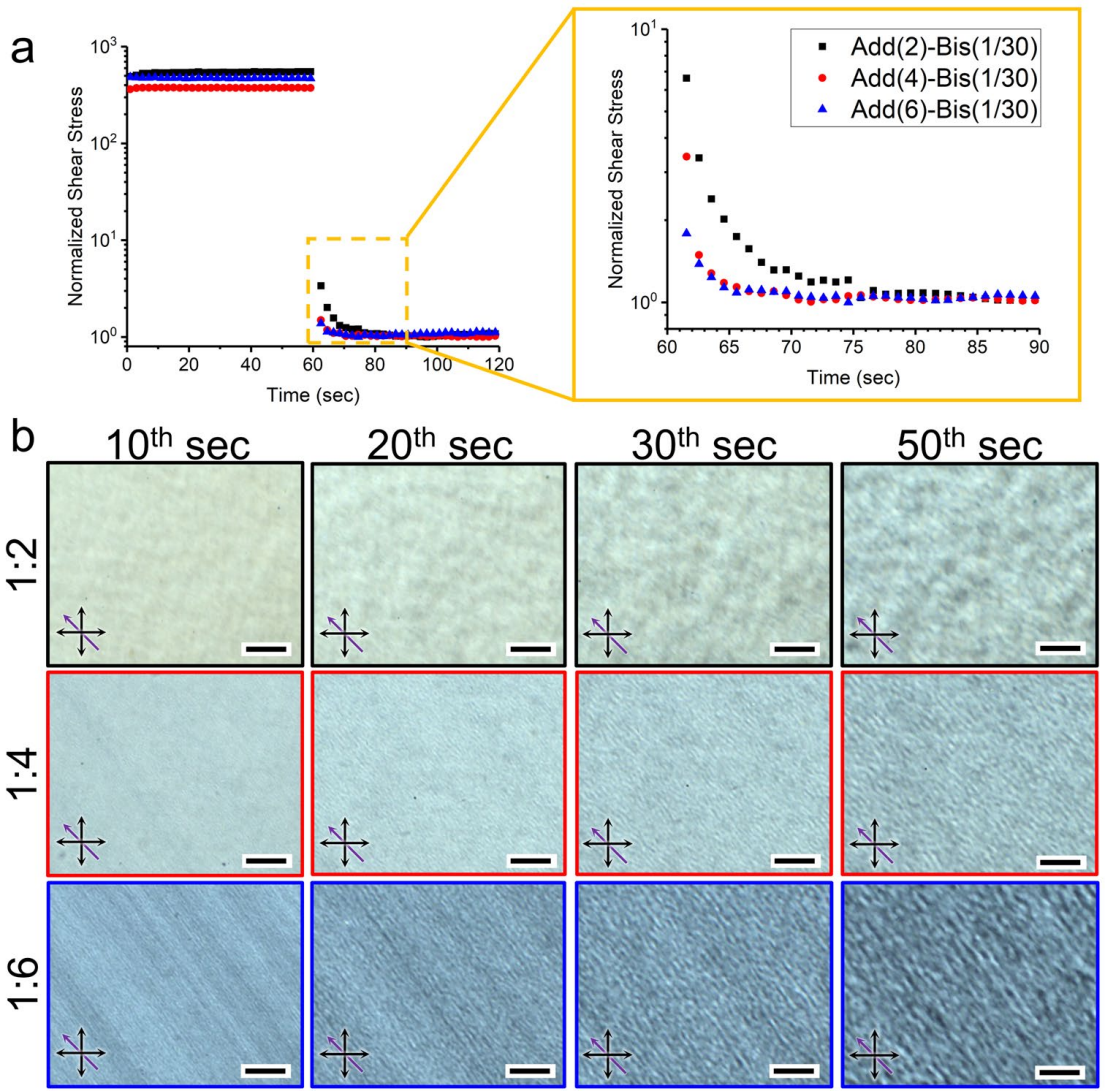
The stress and structural relaxation within the first minute after shear removal. **a** The shear stress relaxation of various compositions over the transient step of decreasing the shear rate from 37 to 0.01 s^−1^ at the 60th second. For comparison purposes, plots are normalized to their minimum values. **b** POM time series of the structural relaxation of different inks within the first minute after removing the rotational flow of 37 s^−1^. Black and purple arrows indicate the polarizer/analyzer and the flow direction, respectively. Scale bars are 100 µm

During the DIW process, the deposited filaments undergo a photo-curing process via exposure to UV light. This photo-curing leads to the creation of a polymer network that arrests any further structural changes. Due to the rapid nature of the photo-curing reaction, it is necessary to achieve recovered chiral structures before the structure becomes arrested within the cross-linked network of AAm and Bis. In order to evaluate the chiral relaxation within the first few seconds after the flow cessation, we face experimental challenges in our in situ microfluidic approach due to the limited resolution of the optical microscopy as well as the low pitch length values of recoiling structures at the earlier times (Figs. 3c and S2). Instead, we conducted transient rotational rheometry to simulate the flow conditions that inks experience after deposition. To achieve this, we suddenly decreased the steady-state shear rate from 37 s^−1^ (the average shear rate in our DIW process) to 0.01 s^−1^ and monitored the viscous response to the transient deformation (Fig. 4a). The shear rate of 0.01 s^−1^ is chosen to maintain shear stresses within the measuring range of the rheometer while ensuring the formation of chiral nematic structures within regime I (Fig. 1). In all samples, the shear stress values reach the plateau within 20 s after the transient drop (Fig. 4a, left), while the rate of stress relaxation is observed to be faster as CNC:Add. ratio decreases (Fig. 4a, right). Consistent with the previous studies [34, 47], these measurements confirm that the shear stress relaxes quickly and has no effect on the subsequent structural relaxation within the first minute after the flow cessation. To capture the chiral reformation dynamics within the first minute after the flow cessation, we employed the Linkam optical rheology stage. This allowed us to acquire time-series POM images after pre-shearing samples at an average DIW shear rate of 37 s^−1^. Prior to the flow cessation, all samples displayed distinct birefringence patterns characterized by bright regions, similar to those observed at high shear rates in Fig. 1d. Consistent with the in situ microfluidic analysis (Fig. 3), chiral nematic structures relaxed faster as the monomer content increased (Fig. 4b). In sample Add(2)-Bis(1/30), the earliest fingerprint patterns were detectable approximately 30 s after flow cessation, while in samples Add(4)-Bis(1/30) and Add(6)-Bis(1/30), they were visible at the 20th and 10th second, respectively (Fig. 4b). Further relaxation proceeded with expansion of the helical assembly and enlarging the pitch lengths. This methodology allows us to identify the minimum time required for the recovery of chiral nematic structures before the gelation of reactive inks.

### Kinetic of Photo-Polymerization in Chiral Nematic Inks

**Fig. 5:**
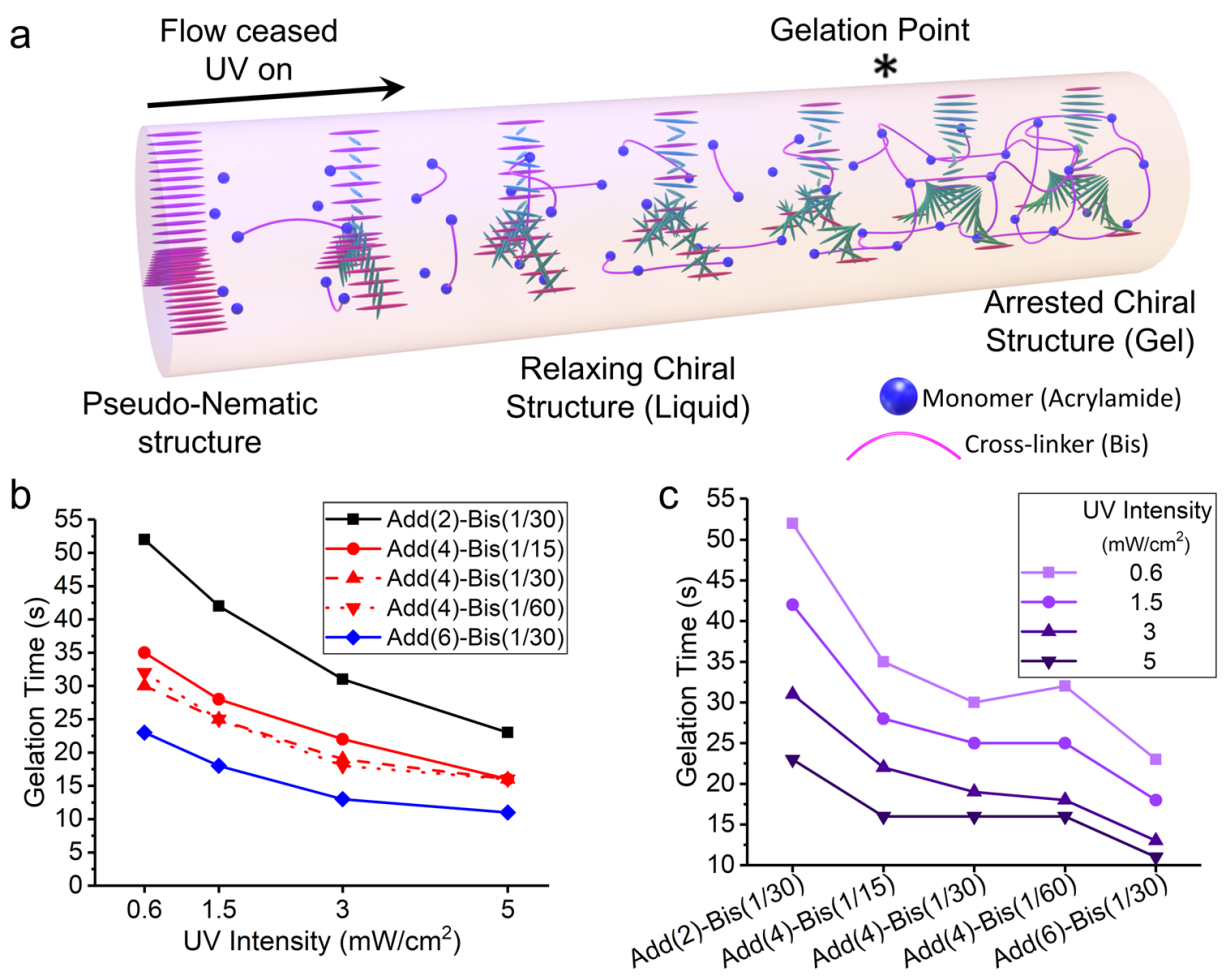
Extracted gelation time values from the UV curing rheometry experiments. **a** A schematic illustration of the gelation process under UV exposure, which locks in the recovered chiral nematic structure within the cylindrical confinement of printed filaments. **b** Gelation time as a function of UV intensities for different CNC:Add. and Bis:AAm. Ratios. **c** Gelation time as a function of composition at different UV intensities

In order to create self-supporting filaments after printing, a cross-linked network of AAm and Bis must be formed. Moreover, the kinetics of the cross-linking reactions defines how much time the chiral nematic structure has to recover before reaching the gelation point, where G′ > G′′ (elastic, G′, and loss, G′′, moduli) (Fig. 5a). To determine the gelation time, we used the UV curing rheometry technique. This involves the UV light illumination during oscillatory measurements within the linear viscoelastic region (1 rad s^−1^ of angular frequency and 0.1% of strain) at the 300th second and taking the cross-over of G′ and G′′ as the gelation time (Fig. S5a-e). This method is widely adopted for evaluating the curing behavior of photo-reactive materials in 3D printing [26, 62–66]. We examined the impact of various factors such as UV intensity, photo-curable monomer content, and cross-linker:monomer ratio (Bis:AAm) on the photo-polymerization kinetics and the gelation time of the chiral inks (Fig. S5a–e). Our results indicate that higher UV intensities during photo-polymerization lead to a shorter gelation time, reducing the available time for the chiral structure to recover for a given CNC:Add ratio (Fig. 5b). Moreover, as the additive content increases, the decrease in gelation time with UV intensity becomes less significant. This can be attributed to the higher concentration of available free radicals within the system (Fig. 5b), which results in a narrower time window for gelation. For instance, in sample Add(2)-Bis(1/30) with the lowest monomer content, increasing the UV intensity from 0.6 to 5 mW cm^−2^ resulted in a 29-s reduction in gelation time (from 52 to 23 s, as shown in Fig. 5b). On the other hand, in sample Add(6)-Bis(1/30) with the highest monomer content, the gelation time decreased by 12 s (from 11 to 23 s, Fig. 5b) when the UV intensity was increased to 5 mW cm^−2^. At a given UV intensity, an observed trend also showed a decrease in gelation time when the concentration of photo-curable monomers was increased. (Fig. 5c). However, at higher UV intensities, the descending rate was reduced, indicating that the formation of the cross-linked network of AAm and Bis is relatively quick at any given composition (Fig. 5b).

We further investigated the influence of the cross-linker ratio (Bis:AAm) on the gelation time, focusing on the chiral inks with a CNC:Add ratio of 1:4 (coded with Add(4)). Gelation time was measured for chiral inks with Bis:AAm ratios of 1/15, 1/30, and 1/60. The results indicated that the gelation time remained relatively constant regardless of the cross-linker ratio (Fig. 5b, c). This implies that the cross-linker ratio has a negligible effect on the kinetics of the photo-polymerization process, unlike the monomer amount, which had a significant impact (Fig. 5b, c). Thus, it appears that the primary governing parameters of gelation kinetics are the UV intensity and the AAm concentration.

### Effect of UV Intensity and Composition on Printed Filaments

**Fig. 6:**
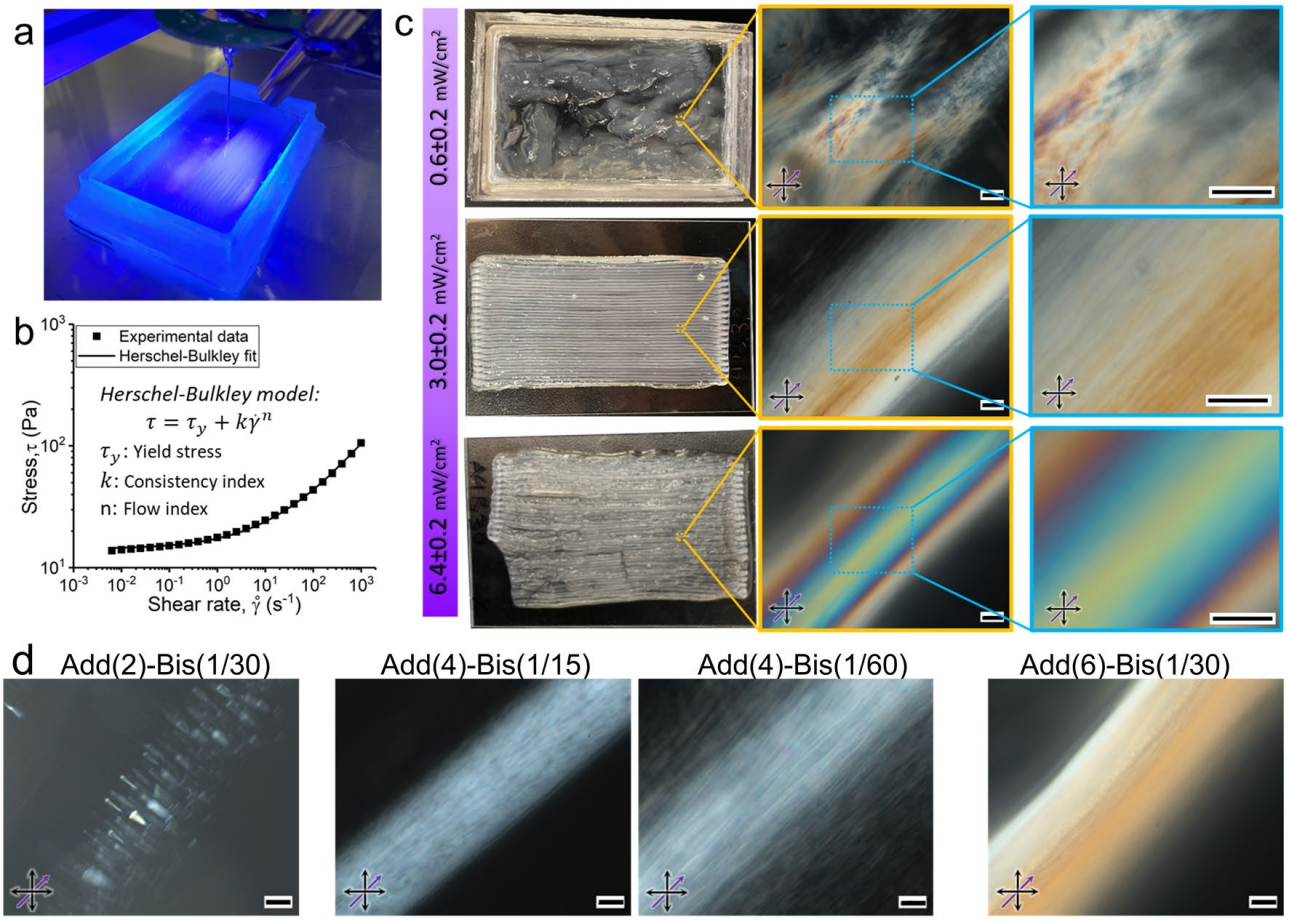
Effect of UV intensity and composition on DIW quality and internal structure. **a** Employed DIW approach of photo-curable inks within Carbopol microgels, as supporting baths, under continuous UV exposure. **b** Shear flow profile of Carbopol microgel at the concentration of 0.50 wt% and fitted Herschel-Buckley model. **c** The left column shows the photographs of printed sheets using ink Add(4)-Bis(1/30) in the rectilinear pattern under different UV intensities after were pulled out from supporting baths. Two columns on right depict their corresponding POM images. **d** POM images of the printed filaments using inks Add(2)-Bis(1/30), Add(6)-Bis(1/30),Add(4)-Bis(1/15), and Add(4)-Bis(1/60) at the UV intensity of 3.0 mW cm^−2^. Samples were pulled out of the supporting bath and were fully swelled in water before taking POM images. Black and purple arrows indicate the polarizer/analyzer and the flow direction, respectively. Scale bars are 100 µm

The incorporation of reactive additives into the low-viscous and liquid-like CNC-based inks aims to improve the shape-fidelity of printed filaments during the photo-polymerization process. As photo-polymerization initiates after filament deposition, extruded inks require a supporting bath to retain the printed filament until the polymer network formation is complete (Fig. 6a). In this study, we used jammed Carbopol microgels as a liquid-like solid (LLS) bath, which is commonly used in embedded ink writing (EIW) [34, 68–72]. Jammed granular microgels of Carbopol, as a common carbomer, follow Herschel-Bulkley rheological behavior, that liquefy under shear experienced in front of the nozzle and retain their solid-like structure when the shear force is removed [73, 74]. Moreover, their non-thixotropic property ensures that the microgels yielded at the nozzle's tip rapidly regain their solid-like state [75], thereby preventing them from flowing back into the ink-filled channels. However, the wake created behind the nozzle must be refilled by the surrounding medium, which necessitates the yielding of the surrounding microgel under the hydrostatic pressure of Carbopol microgels above the nozzle's tip ($\tau_{y} \ge \rho gh$) [71]. Thus, the depth of the embedded printing and the yielding stress of Carbopol microgels must be determined carefully to ensure optimum EIW conditions. In this study, a concentration of 0.50 wt% of Carbopol microgels provided sufficient yielding stress ($\tau_{y} = 13.75 {\text{Pa}}$, Fig. 6b) for EIW at the printing depth of at least 6 mm.

We subsequently investigated the effect of UV intensity, monomer concentration and crosslink ratio on the printing quality and the microstructure of the printed filaments within the supporting bath. To do so, we first studied the effect of UV intensity for the chiral ink Add(4)-Bis(1/30). When polymerized at low UV intensity of 0.6 mW cm^−2^, the printed filaments lacked structural integrity due to the prolonged gelation time (> 30 s, see Fig. 5c) and intermixing with the surrounding aqueous microgel (Fig. 6c, top row). In contrast, at a high UV intensity of 6.8 mW cm^−2^, the short gelation process (< 15 s, see Fig. 5c) surpassed the chiral reformation (~ 20 s, see Figs. 3c, 4b, and S2) and resulted in the formation of flow-induced birefringence patterns that correspond to the nematic alignment of the CNC particles (POM image in Fig. 6c, bottom row). Moreover, the fast gelation process develops elastic instabilities and shape irregularities in the printed filaments, deteriorating the printing quality (Fig. 6c, bottom row). At the UV intensity of 3.0 mW cm^−2^, uniformly aligned fingerprint patterns were detected in the POM image of the polymerized filaments (Fig. 6c, middle row), indicating a good balance between the gelation (~ 25 s, see Fig. 5c) and the chiral relaxation phenomena (~ 20 s, see Figs. 3c, 4b, and S2). The filaments also showed adequate self-supporting features, with no visible nonuniformity. Therefore, we consider this UV intensity to be optimal and used it to examine the effect of monomer content and cross-linker ratio on the printed filaments.

At the optimum UV intensity of 3.0 mW cm^−2^, we further examined the impact of reactive monomer concentration on the printing quality and microstructure of the polymerized filaments.

All prints were carried out in Carbopol microgel supporting baths, while maintaining a constant crosslinker to monomer (Bis:AAm) ratio of 1:30. When printing with the lowest monomer content, the resulting filaments exhibited an exceptionally soft with distorted fingerprint patterns (sample Add(2)-Bis(1/30) in Fig. 6d). These observations indicate that a minimum concentration of monomers is required to achieve successful printing of self-supporting materials. In sample Add(6)-Bis(1/30), on the other hand, sufficient density of polymeric network impedes the integrity deficiencies (Fig. 6d). Despite the short gelation time of this sample at the UV intensity of 3.0 mW cm^−2^ (13 s, see Fig. 5), the fast chiral relaxation process (~ 10 s after the flow cessation in Figs. 3c, 4b, and S2) ensured the recovery of the chiral nematic structure before the gelation time.

To study the effect of cross-linking density (i.e., Bis:AAm ratio) on the printing quality and arrested chiral structures, we varied the Bis:AAm ratio among 1:15, 1:30, and 1:60 at the constant CNC:Add. ratio of 1:4 (samples Add(4)-Bis(1/15), Add(4)-Bis(1/30), and Add(4)-Bis(1/60), respectively). The rheological measurements of these inks indicated that their gelation time remained largely unaffected by variations in the cross-linker amount present in the inks (Fig. 5). However, altering the cross-linking density led to a notable difference in the swelling behavior of the polymerized printed structures in the fully hydrated state. As the Bis:AAm ratio decreased from 1:15 to 1:60, polymerized filaments with a looser polymer network were formed, which could swell to a greater extent, leading to the expansion of the characteristic pitch length (Fig. 6d). This suggests that the characteristic pitch length of the arrested chiral nematic structure can be selectively adjusted in the fully hydrated state by modifying the cross-linking density, without impacting the chiral relaxation or gelation kinetics.

**Fig. 7:**
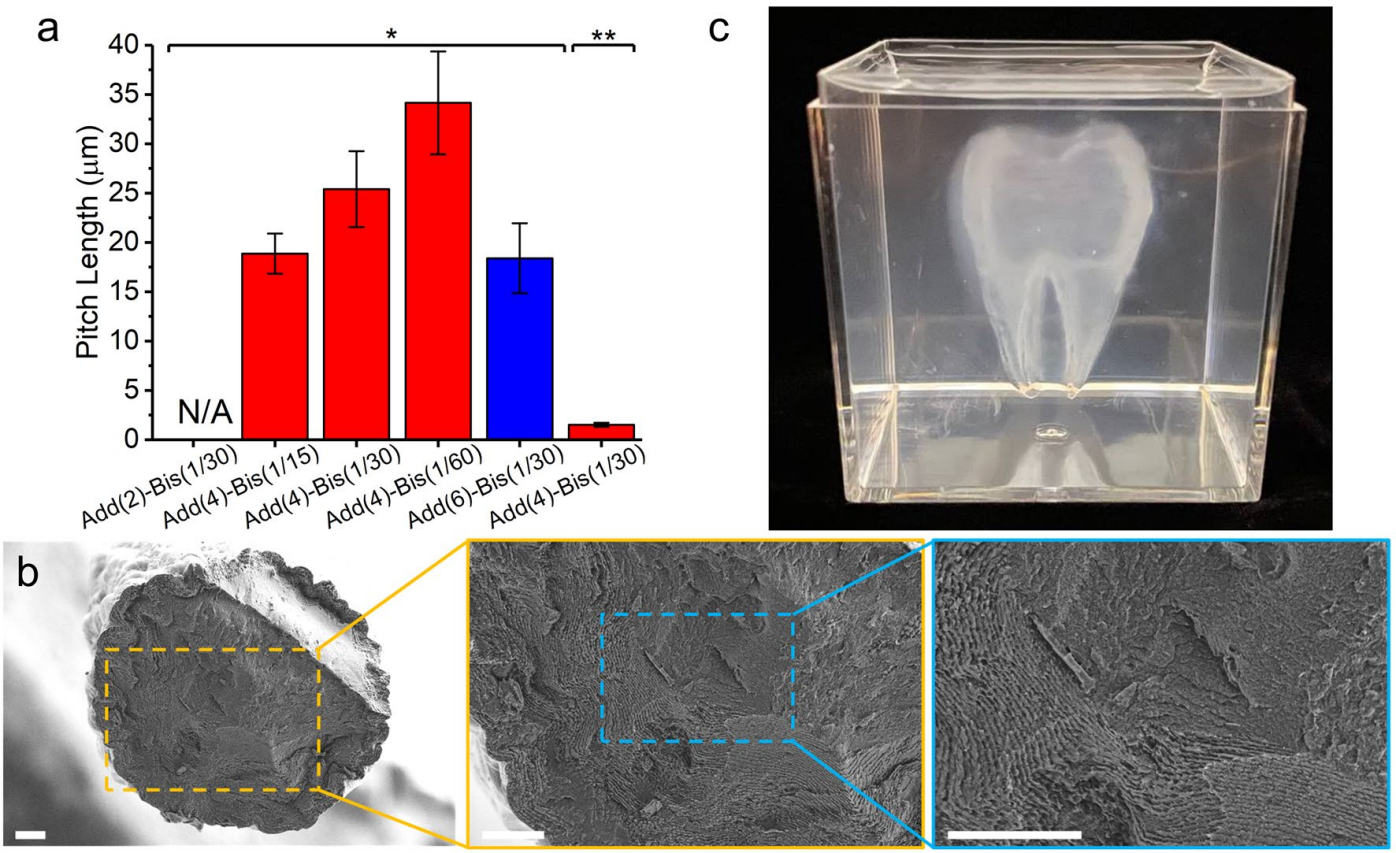
**a** Pitch length value of the printed filaments in the hydrated state (labeled with *) and in the dried state (**). **b** SEM micrograph of the fractured surface of a filament printed using ink Add(4)-Bis(1/30). Scale bars are 20 µm. **c** 3D-printed tooth model within a supporting bath using ink Add(4)-Bis(1/30) under optimal printing conditions

The pitch length of the printed filaments at different ink compositions was determined from the fingerprints of the chiral arrangements observed in POM images (Fig. 6c, d). The ink Add(2)-Bis(1/30), however, resulted in irregular printed filaments, making it challenging to ascertain a consistent pitch length. For the other samples, the characteristic pitch length ranged from 18 to 34 µm (Fig. 7a). Beyond confirming the aligned chiral structure along the print direction using POM, we have also verified the 3D concentric arrangement of chiral assembly in the transverse cross-section via SEM micrographs (Fig. 7b). A fractured surface of a single filament printed with ink Add(4)-Bis(1/30) exhibits radial fingerprint patterns (Fig. 7b). It is important to note that some distortions are inevitable due to the significant contraction of the printed hydrogels during the drying process, causing an uneven fractured surface (Fig. 7b). Therefore, the pitch length of the dried filaments was measured at various locations. For the dried filament using the ink Add(4)-Bis(1/30), the average pitch length was found to be about 1.5 µm (Fig. 7a).

Overall, the outcomes confirm that the final spatial arrangement of CNC nanoparticles and the resolution of the chiral hierarchy within the printed constructs can be engineered by tuning the reactive ink components, 3D printing conditions, and photo-polymerization kinetics. Furthermore, the optimized printing conditions can be extended to design intricate 3D geometries with built-in chiral nano/microstructures. To demonstrate this capability, a tooth model was 3D printed using ink Add(4)-Bis(1/30) within the supporting bath (Fig. 7c).

## Conclusion

This work presented a novel strategy for 3D printing, providing control over the chiral self-assembly pathways in reactive chiral nematic inks. These inks, designed using cellulose nanocrystal (CNC) particles and photo-curable components like acrylamide (AAm) and N,N′-methylenebisacrylamide (Bis), allow the creation of structures that leverage chiral hierarchies at the nano- and microscales. The strategy, unlike traditional approaches, harnesses the potential of 3D printing in manipulating soft matter, going beyond the limitations of sub-voxel microstructures commonly seen in printed materials.

The core of our approach revolves around a deep understanding of the behavior of designed reactive inks. This includes studying the effects of shear flow on chiral assembly within the DIW nozzle, the dynamics of chiral relaxation post flow cessation, the photo-curing kinetics under varied UV intensities, and reactive contents, as well as assessing the print quality of embedded printed filaments.

By utilizing a range of complementary techniques such as orthogonal superposition rheology, optical shear rheometry, and microfluidics combined with optical microscopy, we have been able to scrutinize the structural evolution of these inks. Our study reveals an intricate phase transformation of the chiral assembly under shear, transitioning from tilting chiral domains to pseudo-nematic structures. These insights into the rheology and the self-assembly behavior of CNCs mark a significant advance in the field, expanding our understanding of how to use shear flow and photo-polymerization kinetics to control the pitch length of chiral structures at nano/micro scales.

This study has introduced an engineered strategy that not only enables 3D printing of bio-inspired intricate structures with uniformly aligned chiral nematic structures but also allows for precise control over the chiral self-assembly pathway during the print processing at sub-voxel scales. By integrating the process of 3D printing into the design of materials and understanding the interplay of flow-chirality interactions and the photo-curing induced gelation, we have increased the flexibility and potential of current 3D printing techniques. This could pave the way for the development of advanced materials in numerous areas such as biomaterials, photonics, sensors, shape-morphing materials, and tissue engineering.

## Supplementary Information

Below is the link to the electronic supplementary material.

Supplementary file1 (PDF 845 kb)
