# Supplementary material for: Engineering Nano/Microscale Chiral Self-Assembly in 3D Printed Constructs
Source: Nanomicro Lett. 2023 Dec 18;16:54. doi: 10.1007/s40820-023-01286-0 (PMC10728402; doi:10.1007/s40820-023-01286-0)
Supplement: Supplementary file 1 — Supplementary file1 (PDF 845 kb) [file 40820_2023_1286_MOESM1_ESM.pdf]

Supporting Information

Engineering Nano/Microscale Chiral Self-Assembly in 3D Printed Constructs

Mohsen Esmaeili[1], Ehsan Akbari[2], Kyle George[1], Gelareh Rezvan[1], Nader Taheri-Qazvini[1, 3], Monirosadat Sadati[1, *]

[1]Department of Chemical Engineering, University of South Carolina, Columbia, SC, 29208, United States

[2]TA Instruments, Waters LLC, New Castle, DE, 19720, United States

[3] Biomedical Engineering Program, University of South Carolina, Columbia, SC, 29208, United States

*Corresponding author. E-mail: sadati@cec.sc.edu (Monirosadat Sadati)

S1 Average Shear Rate

To calculate the average shear rate within the 3D printer's nozzle, we employed the equation for $\dot{\gamma}_{ave.} = \frac{3n+1}{2n+1}\frac{2V_{ave.}}{r}$, where n, $V_{ave.}$, and r are power law index, average flow velocity (3 mm/sec), and the nozzle radius (205 µm), respectively [S1, S2]. This n value is determined as an average obtained from fitting the power law model to flow curve of three ink compositions at the second shear thinning regions (regime III) (shear rates from 10 to 1,000 s^{-1}), as illustrated in Fig. S1. The fitted power law indices for inks Add(2)-Bis(1/30), Add(4)-Bis(1/30), and Add(6)-Bis(1/30) are 0.53, 0.63, and 0.73, respectively. Consequently, the average power law index, n, is computed as 0.63. Using this value, the average shear rate experienced by the inks within the nozzle of the 3D printer during DIW was calculated to be 37 s^{-1}.

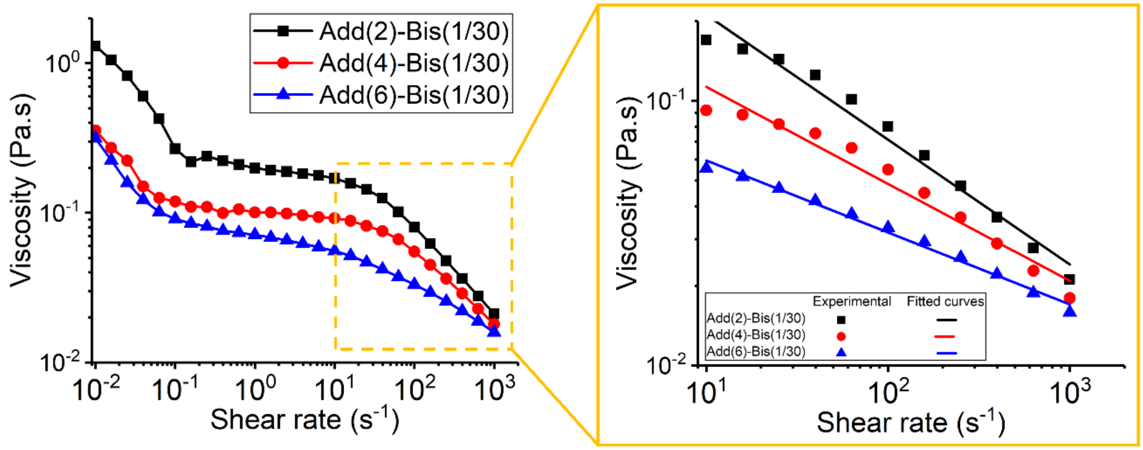

Fig. S1 Viscosity plots as a function of rotational shear rate for different inks at a constant monomer:cross-linker ratio of 1:30. The inset shows the second shear thinning region, regime III, where the power law model is fitted to calculate power law indices

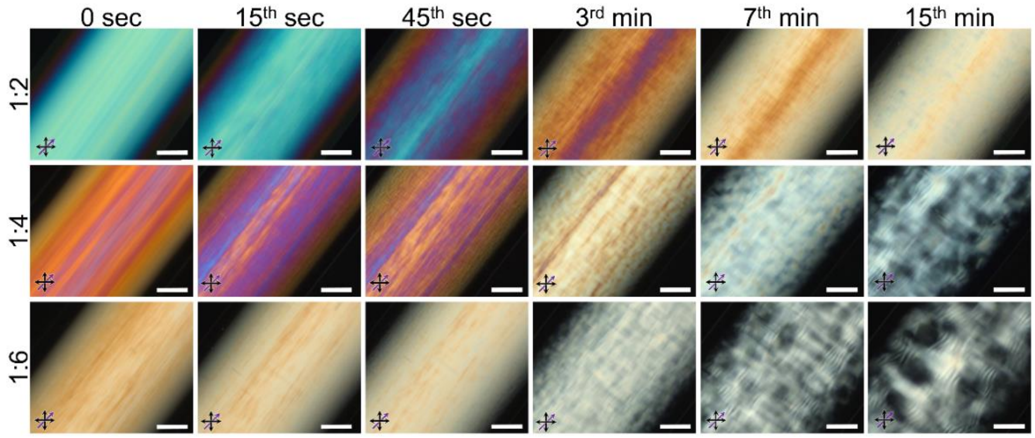

Fig. S2 Time-series snapshots of the structural evolution after the flow cessation of inks with different CNC:Add. raios after the flow cessation under POM, using a white light source. Black and purple arrows indicate the polarizer/analyzer and the flow direction, respectively. Scale bars are 100 µm

We coupled a lab-built microfluidic setup containing a quartz cylindrical capillary and a polarized optical microscope to investigate the structural evolution of chiral inks under conditions that resemble the shear flow in the DIW nozzle (Figure 3a) [S3]. This setup, featuring a capillary tube with a diameter of DIW's nozzle (approximately 400 µm), aligned at 45° with respect to the cross-polarizer axis, allowed us to directly visualize the structural changes of the CNC-based inks at a flow rate equivalent to that of the DIW process in this study (i.e., 0.37 µL/sec) [S3]. Flow-induced birefringence patterns are obtained by using a white light source in the cross-polarized configuration. For birefringence measurements, POM images were taken using a monochromic light, filtered by a 500 nm bandpass light filter (FB500-10, Thorlabs, λ=500 nm) in two configurations of cross- and parallel-polarizers. Corresponding light intensities in cross- and parallel-polarized monochromic images ($I_\parallel$, and $I_\perp$, respectively) as a function of radial position were measured using ImageJ, and input in the following equations to find retardation phase values, δ [S4]:

$$\delta = N\pi + 2\tan^{-1}\sqrt{\frac{I_\perp}{I_\parallel}}, N = 0,2,4, \ldots \qquad \text{(S1)}$$

$$\delta = (N+1)\pi - 2\tan^{-1}\sqrt{\frac{I_\perp}{I_\parallel}}, N = 1,3,5, \ldots \qquad \text{(S2)}$$

Where N is a non-negative integer that corrects the order of retardation lengths, arising from the periodic functions of light intensities. Therefore, the birefringence value, Δn, was calculated using Eq. (3):

$$\Delta n = \frac{\delta\lambda}{2\pi d} \qquad \text{(S3)}$$

Where λ is the wavelength of the monochromic light (i.e., 500 nm), and d is the sample's thickness. By observing the transmitted cross-polarized light, the flow-induced alignment generates iridescent colors of retarded light (Fig. S3a), which can be directly correlated to the colormap of the Michel-Levy chart [S5]. To quantify the flow-induced birefringence features, we measured monochromic light intensities at two configurations of cross- and parallel polarized light (**Fig.** S3b) as a function of radial position and converted them into the retardation length, Γ, using equations

(1), and (2) (Fig. S3c). The calculated retardation lengths exhibit close agreement with the observed colors in Fig. S3a when overlaid with the Michel-Levy chart cut in the background. As the retarded light was perpendicularly transmitted through capillary tubes with varying thicknesses, retardation lengths are inherently a function of thickness. Additionally, birefringence values, Δn, were determined from the retardation length values using equation (3) and plotted as a function of the radial position (Fig. S3d). We observe an exponential growth in Δn as we move away from the centerline. The maximum Δn is typically observed at the wall due to the surface anchoring effect and the highest degree of shear-induced alignment (Fig. S3d). Increasing the additive and thereby reducing the CNC in the CNC-based inks shifts the retardation length and Δn curves to lower values [S3, S6].

In our previous study [S3], simulation results indicated a high Erickson number, Er, of 24,000 in these experiments ($Er = \frac{\eta V d}{K}$, where η is the viscosity at Newtinain plateau regime (regime II), V is the equivalent flow velocity, d is the capillary's diameter, and K is the elastic constant equal to 10 pN). This suggests that the pseudo-nematic structure is driven by a strong flow-aligning effect inside the DIW's nozzle. The formation of the pseudo-nematic structure can also be identified based on the fully iridescent POM images with the maximum intensity at 45° with respect to the analyzer/polarizer direction, revealing the orientation of liquid crystalline director fields along the flow direction. These measurements offer qualitative data on the extent of shear-induced alignment of CNC particles in the 3D printer's nozzle and serve as the initial state for the subsequent chiral relaxation dynamics discussed in the main manuscript.

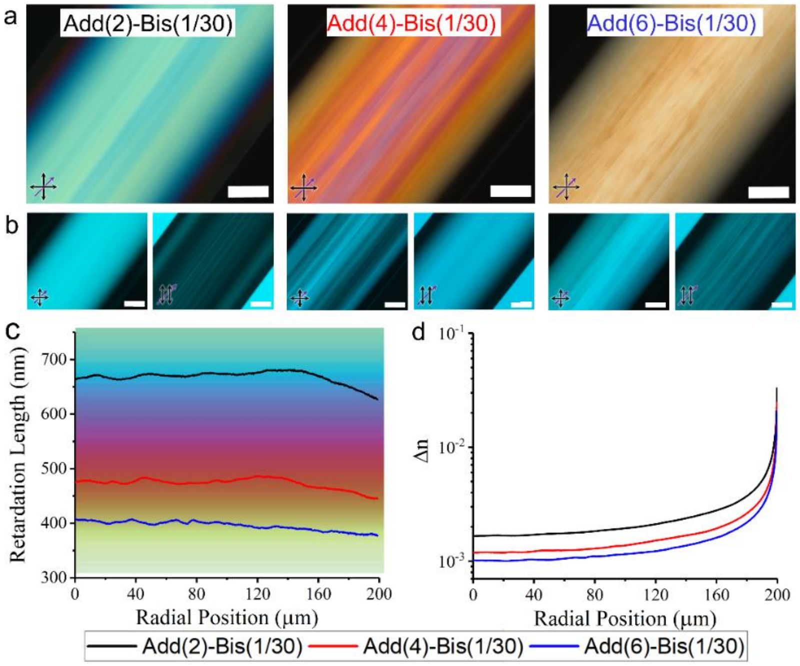

Fig. S3 flow-induced alignment of CNC particles under Poiseuille flow. (**a**) cross-polarized birefringence patterns of various inks under Poiseuille flow through quartz capillary tubes with an inner diameter of 400 μm using white light source. (**b**) cross-polarized and parallel-polarized micrographs of flow in the capillary tube using a monochromic light source (λ=500 nm). (**c**) Retardation length values, Γ, and (**d**) Birefringence values, Δn, as a function of radial position Black and purple arrows indicate the polarizer/analyzer and the flow direction, respectively. Scale bars are 100 μm

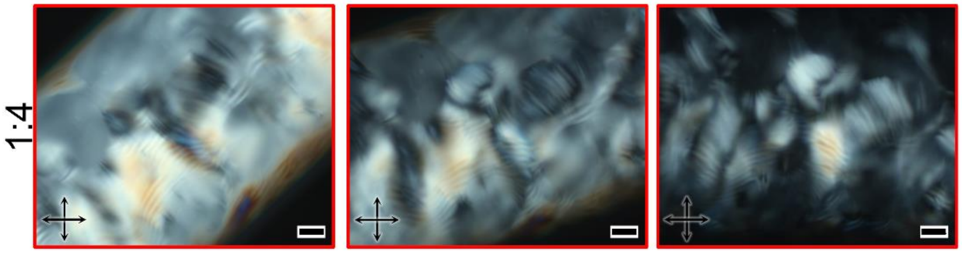

Fig. S4 POM images of capillary tubes filled with different CNC-based inks one hour after flow cessation at different angles of flow direction with respect to the polarizer direction. Scale bars are 50 μm

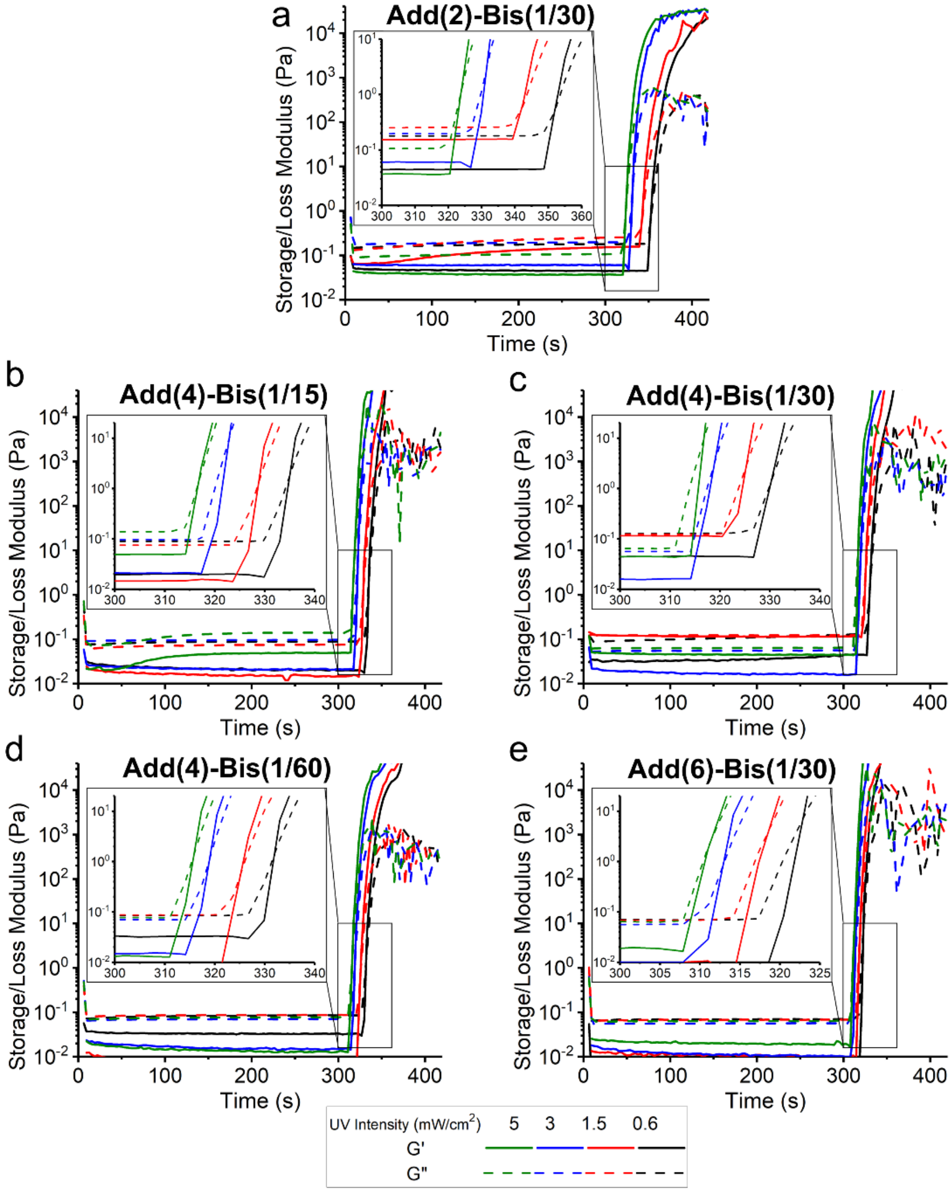

Fig. S5 UV rheometry photo-curable inks. Storage, G', and loss, G", moduli as a function of time for samples (**a**) Add(2)-Bis(1/30), (**b**) Add(4)-Bis(1/15), (**c**) Add(4)-Bis(1/30), (**d**) Add(4)-Bis(1/60), and (**e**) Add(6)-Bis(1/30), at different UV intensities, which set on at the 300th second

Supplementary References

[S1] F. X. Tanner, A. A. Al-Habahbeh, K. A. Feigl, S. Nahar, S. A. K. Jeelani, et al., Numerical and experimental investigation of a non-newtonian flow in a collapsed elastic tube. Appl. Rheol. **22**, 639101–639108 (2012). https://doi.org/10.3933/ApplRheol-22-63910

[S2] C. Petrie, Dynamics of polymeric liquids. Volume 1. Fluid mechanics (2nd ed). (1988). https://doi.org/10.1016/0377-0257(88)80020-x. https://doi.org/10.1016/0377-0257(88)80020-x

[S3] M. Esmaeili, K. George, G. Rezvan, N. Taheri-Qazvini, R. Zhang, et al., Capillary Flow Characterizations of Chiral Nematic Cellulose Nanocrystal Suspensions. Langmuir **38**, 2192–2204 (2022). https://doi.org/10.1021/acs.langmuir.1c01881

[S4] S.-T. Wu, U. Efron, L. D. Hess, Birefringence measurements of liquid crystals. Appl. Opt. **23**, 3911 (1984). https://doi.org/10.1364/ao.23.003911

[S5] B. E. Sørensen, A revised Michel-Lévy interference colour chart based on first-principles calculations. Eur. J. Mineral. **25**, 5–10 (2013). https://doi.org/10.1127/0935-1221/2013/0025-2252

[S6] M. Alizadehgiashi, A. Khabibullin, Y. Li, E. Prince, M. Abolhasani, et al., Shear-Induced Alignment of Anisotropic Nanoparticles in a Single-Droplet Oscillatory Microfluidic Platform. Langmuir **34**, 322–330 (2018). https://doi.org/10.1021/acs.langmuir.7b03648
